# Mapping Epileptic Networks Using Simultaneous Intracranial EEG-fMRI

**DOI:** 10.3389/fneur.2021.693504

**Published:** 2021-09-21

**Authors:** Umair J. Chaudhary, Maria Centeno, David W. Carmichael, Beate Diehl, Matthew C. Walker, John S. Duncan, Louis Lemieux

**Affiliations:** ^1^Department of Clinical and Experimental Epilepsy, University College London (UCL) Institute of Neurology, National Hospital for Neurology and Neurosurgery, London, United Kingdom; ^2^Magnetic Resonance Imaging (MRI) Unit, Epilepsy Society, Chalfont St. Peter, United Kingdom; ^3^Neurology Department, University Hospital Coventry and Warwickshire, Coventry, United Kingdom; ^4^Epilepsy Unit, Neurology Department, Hospital Clinic Barcelona, Barcelona, Spain; ^5^Imaging and Biophysics Unit, University College London (UCL) Institute of Child Health, London, United Kingdom; ^6^Clinical Neurophysiology, National Hospital for Neurology and Neurosurgery, London, United Kingdom

**Keywords:** intracranial EEG, EEG-fMRI, IED/spikes, BOLD, post-surgical outcome

## Abstract

**Background:** Potentially curative epilepsy surgery can be offered if a single, discrete epileptogenic zone (EZ) can be identified. For individuals in whom there is no clear concordance between clinical localization, scalp EEG, and imaging data, intracranial EEG (icEEG) may be needed to confirm a predefined hypothesis regarding irritative zone (IZ), seizure onset zone (SOZ), and EZ prior to surgery. However, icEEG has limited spatial sampling and may fail to reveal the full extent of epileptogenic network if predefined hypothesis is not correct. Simultaneous icEEG-fMRI has been safely acquired in humans and allows exploration of neuronal activity at the whole-brain level related to interictal epileptiform discharges (IED) captured intracranially.

**Methods:** We report icEEG-fMRI in eight patients with refractory focal epilepsy who had resective surgery and good postsurgical outcome. Surgical resection volume in seizure-free patients post-surgically reflects confirmed identification of the EZ. IEDs on icEEG were classified according to their topographic distribution and localization (Focal, Regional, Widespread, and Non-contiguous). We also divided IEDs by their location within the surgical resection volume [primary IZ (IZ1) IED] or outside [secondary IZ (IZ2) IED]. The distribution of fMRI blood oxygen level-dependent (BOLD) changes associated with individual IED classes were assessed over the whole brain using a general linear model. The concordance of resulting BOLD map was evaluated by comparing localization of BOLD clusters with surgical resection volume. Additionally, we compared the concordance of BOLD maps and presence of BOLD clusters in remote brain areas: precuneus, cuneus, cingulate, medial frontal, and thalamus for different IED classes.

**Results:** A total of 38 different topographic IED classes were identified across the 8 patients: Focal (22) and non-focal (16, Regional = 9, Widespread = 2, Non-contiguous = 5). Twenty-nine IEDs originated from IZ1 and 9 from IZ2. All IED classes were associated with BOLD changes. BOLD maps were concordant with the surgical resection volume for 27/38 (71%) IED classes, showing statistical global maximum BOLD cluster or another cluster in the surgical resection volume. The concordance of BOLD maps with surgical resection volume was greater (*p* < 0.05) for non-focal (87.5%, 14/16) as compared to Focal (59%, 13/22) IED classes. Additionally, BOLD clusters in remote cortical and deep brain areas were present in 84% (32/38) of BOLD maps, more commonly (15/16; 93%) for non-focal IED-related BOLD maps.

**Conclusions:** Simultaneous icEEG-fMRI can reveal BOLD changes at the whole-brain level for a wide range of IEDs on icEEG. BOLD clusters within surgical resection volume and remote brain areas were more commonly seen for non-focal IED classes, suggesting that a wider hemodynamic network is at play.

## Introduction

Intracranial electroencephalography (icEEG) recordings are performed during presurgical evaluation to localize irritative zone (IZ), seizure onset zone (SOZ), epileptogenic zone (EZ), and eloquent cortex for patients being considered for epilepsy surgery. icEEG has better spatial resolution and sensitivity compared to scalp EEG (1), which has low sensitivity (2, 3) and can provide inaccurate localization (4) and even lateralization (5), especially in patients with frontal lobe epilepsy. icEEG, however, has limited spatial sampling, only detecting electrical activity within a 1-cm core of tissue from recording site (6), and carries surgical risk (7, 8).

Simultaneous scalp EEG and functional magnetic resonance imaging (EEG-fMRI) can map interictal epileptiform discharges (IED) and seizure-related blood-oxygen-level-dependent (BOLD) changes (9–21). In patients with focal cortical dysplasia, IED-related BOLD changes distributed over multiple lobes are associated with poor postsurgical outcome (22). The interpretation of EEG-fMRI findings is often limited by the low sensitivity of scalp EEG, low rates of IED, and an incomplete understanding of the relationship between IED and BOLD changes (12, 13, 23–25). The sensitivity of EEG-fMRI can be increased by using topographical map correlation-based comparison of EEG recorded inside and outside the scanner (26). Simultaneous icEEG and fMRI (icEEG-fMRI) has been performed following extensive safety testing and locally adapted protocol (27–30) [see (31) for review], revealing IED-related BOLD changes local and remote from the relevant intracranial electrodes (32, 33).

We used icEEG-fMRI to explore BOLD changes and their distribution at the whole brain level for different IED classes on icEEG, in patients with refractory focal epilepsy who had good postsurgical outcome and well-characterized EZ. Surgical resection volume in this group of patients with good postsurgical represents confirmed identification of the EZ [where EZ is area of the brain deemed necessary to be resected to render patient seizure free (1, 34)].

Our hypotheses were as follows: (1) widespread BOLD networks, involving the surgical resection volume and remote brain areas, can be seen for IEDs on icEEG; (2) distribution of BOLD changes in surgical resection volume and remote brain areas is different for IED classes based on their topographic localization and their relationship with surgical resection volume. We investigated the anatomical localization and level of concordance of IED-related BOLD maps with the surgical resection volume. We also evaluated the relationship between different IED classes and level of concordance of BOLD maps with the surgical resection volume, and different IED classes and presence of BOLD changes in remote healthy cortex and other brain areas.

## Methods

Eight patients with refractory focal epilepsy had icEEG-fMRI during their invasive pre-surgical evaluation, who had subsequently undergone resective epilepsy surgery with a good postsurgical outcome, i.e., completely seizure free or only auras (ILAE class I or II outcome) for more than 2 years after surgery. All patients gave written informed consent. The study was approved by the joint research ethics committee of the National Hospital for Neurology and Neurosurgery, Queen Square, London (UCLH NHS Foundation Trust) and UCL Institute of Neurology, Queen Square, London, UK.

### Clinical Background

Prior to implantation, all patients had undergone detailed clinical history and examination, a structural MRI as per protocol specifically designed for epilepsy (35), long-term scalp video-EEG monitoring, neuropsychological and neuropsychiatric assessments, and additional functional imaging tests including positron emission tomography (PET), magnetoencephalography (MEG), or ictal single photon emission computed tomography (ictal SPECT) as indicated (see Table 1).

**Table 1:** Clinical characteristics.

**ID**	**Age**	**Sex**	**Age @ seizure onset**	**Epilepsy**	**Scalp EEG**	**MRI**	**Other non-invasive investigations**
1	39	M	8	FLE	Sharp: R centro-parietal Seizure: central fast activity	*L HS	PET: R parietal and posterior frontal hypometabolism Ictal SPECT: bi frontocentral and R insular hyperperfusion MEG: R temporo-occipital and frontocentral spikes
2	28	M	12	FLE	Spike: L fronto-central Seizure: regional central	FCD L posterior SFG + MFG	PET: No focal hypometabolism Ictal SPECT: L frontal lobe
3	36	F	7	FLE	Spikes: L inferior frontal/orbito-frontal Seizure: regional L frontal	FCD L IFG	PET: L frontal hypometabolism
4	39	M	9	FLE	Spikes: Regional L temporal-frontal Seizure: Regional L fronto-central	FCD L posterior MFG	PET: No focal hypometabolism
5	32	M	16	FLE	Spikes: Regional R frontal, bi frontal and L fronto-temporal Seizure: Bi frontocentral	NL	PET: R frontal hypometabolism
6	27	F	3	FLE	Spikes: None Seizure: Regional L frontocentral frontocentral	FCD L superior frontal sulci	PET: L SFG hypometabolism Ictal SPECT: L frontal and insular hyperperfusion MEG: no spikes recorded
7	26	M	7	TLE	Spikes: Bi temporal regional Seizure: Regional L temporal	L HS	None
8	28	M	7	PLE	Spikes: Regional R anterior parietal Seizure: Focal R postcentral	FCD Right Supramarginal gyrus	None

M, male; F, female; FLE, frontal lobe epilepsy; TLE, temporal lobe epilepsy; TOLE, Temporo-occipital lobe epilepsy; R, right; L, left; NL, nonlesional; HS, hippocampal sclerosis; FCD, focal cortical dysplasia; SFG, superior frontal gyrus; MFG, middle frontal gyrus; IFG, inferior frontal gyrus; SMC, sensori-motor cortex; HMC, hand motor cortex; *incidental finding.

In accordance with routine clinical practice at our center, implantation of intracranial electrodes was guided by a hypothesis-based consensus decision generated from the results of non-invasive investigations. The SOZ, EZ, and the extent of surgical resection (Table 2) were defined by experienced Clinical Neurophysiologists/Epileptologists (BD, TW, and MW) and members of the multidisciplinary team based on invasive (multiple grid/depth electrode contacts on icEEG) and non-invasive investigations. The implantation scheme for each patient is shown in Supplementary Table 1. Post-surgical outcome (Table 2) was assessed with the ILAE classification (36).

**Table 2:** Invasive localization and post-surgical outcome.

**ID**	**IED type (Number)**	**Spike classification**		**Seizure onset zone**	**Epileptogenic zone**	**Surgical resection**	**Histopathological diagnosis**	**Post-surgical outcome**
		**Topographic distribution**	**Irritative zone (IZ)**					
1	PSMA1–3 (211)	Focal	IZ1: R SMA	R SMA	R SMA and SFG	R SMA and SFG	No FCD confirmed	ILAE II @ 5 years
	ASMA1–3 (46)							
	ASMA1–3 + PSMA1–3 (476)	Regional						
	PC5, 6 (150)	Focal	IZ2: R inferior parietal and MFG					
	PC5, 6 + AI5, 6 (150)	~NC						
2	G4, 5 (72)	Focal	IZ1: L posterior SFG and MFG	L posterior SFG and MFG	L posterior SFG, MFG and SMA	L posterior SFG, MFG and SMA	FCD IIB (Balloon cells present)	ILAE I @ 6 years
	G12–15 (29)							
	G4–6 + G12, 13 + G22–24 + G28–30 (80)	Regional						
	G12–15 + G21–24 + DP2-4 (350)							
	G4–8 + G12–15 + G20–24 + G28–30 + DP2–4 (244)	Widespread						
3	DA3, 4 (770)	Focal	IZ1: L IFG and MFG	L anterior IFG and MFG	L anterior IFG and MFG	L anterior IFG and MFG	FCD IIB (Balloon cells present)	ILAE I @ 9 years
	DA3, 4 + G1 18, 27, 35, 43 (265)	Regional						
	G2 6, 14 (195)	Focal	IZ2: L lateral orbitofrontal					
4	DA3–6 (423)	Focal	IZ1: L IFG and MFG	L inferior MFG	L IFG, MFG and lateral orbitofrontal	L IFG, MFG and lateral orbitofrontal	FCD IIB (Balloon cells present)	ILAE I @ 2 years
	DA4, 5 + GA51 (261)							
	DA2–6 + GA49–54 (208)	Regional						
5	FP2–4 (140)	Focal	IZ1: R anterior inferior orbitofrontal	R anterior IFG and orbitofrontal	R anterior orbitofrontal	R anterior orbitofrontal	No FCD confirmed	ILAE I @ 7 years
	FP2–4 + AM2–4 (44)							
	AM2–4 + FP1–4 PMFG3–6 + IFG9-11 (36: runs of IED lasting 1–9 s)	~NC	IZ2: R anterior inferior orbitofrontal, MFG, IFG, and SMA					
	AM1–4 + FP3–4 FP1–8 + AM1–14 + ASMA2–5+ PMFG3–10 + IFG5–10 (45: runs of IED lasting 3–12 s)							
	FP1–4 + AM1–6 FP1–8 + AM1–14 + FOF1–10 + ASMA2–7 + PMFG4–12 + IFG5–11 (90: runs of IED lasting 3–12 s)							
6	SF5–7 (168)	Focal	IZ1: L SFG (lateral and medial)	L SFG (lateral and medial)	L posterior SFG (lateral and medial)	L posterior SFG (lateral and medial)	FCD IIA (No Balloon cells)	ILAE I @ 7 years
	GB4-6 + 14–16 (90)	Regional						
	GC5–16 (474)							
	SF5–7 + GB5-8 + GC5, 10, 11, 12, 15, 16 (23)	~NC						
7	LAH1, 2 + LPH1, 2 + LA3, 4 (60)	Regional	IZ1: L temporal lobe	SOZ1: L hippocampus SOZ2: R amygdala	L anterior temporal lobe	L anterior temporal lobe	Hippocampal sclerosis No FCD	ILAE I @ 10 years
	LAH1–2 (359)	Focal						
	LA3–4 (57)	Focal						
	LPH 1–2 (96)	Focal	IZ2: R and L temporal lobe					
	RA1, 2 + RH1, 2 (624)	Focal						
	RA1, 2 + RAH1, 2 + LAH2, 3 + LPH2, 3 (10)	Regional						
8	D1+ D2 + G31 (62)	Widespread	IZ1: R supramarginal gyrus	SOZ: R supramarginal gyrus	R supramarginal gyrus extending to hand sensory cortex	R supramarginal gyrus extending to hand sensory cortex	FCD IIB (Balloon cells present)	ILAE I @ 7 years
	D1 3–4 (43)	Focal						
	D2 5–6 (2,481)	Focal						
	G23 (83)	Focal						
	G31 (72)	Focal						
	G36 (209)	Focal						
	G38 (226)	Focal						

*R, right; L, left; NL, non-lesional; HS, hippocampal sclerosis; FCD, focal cortical dysplasia; SFG, superior frontal gyrus; MFG, middle frontal gyrus; IFG, inferior frontal gyrus; SMC, sensori-motor corte; HMC, hand motor cortex; SOZ, seizure onset zone*.

*~Focal/regional with non-contiguous spread*.

### Intracranial EEG-fMRI Acquisition

After the clinical icEEG recordings were completed, the implanted electrodes (numbering between 56 and 128 contacts) were connected to magnetic resonance scanner-compatible cables and amplifier system (32) for icEEG-fMRI acquisition. icEEG was recorded, processed online (to reduce the scanner-related artifacts), and displayed (BrainVision Recorder, Brain Products, Germany) during the fMRI scanning.

In accordance with our icEEG-fMRI protocol (29) echo planar images (EPI: TR/TE/flip angle = 3,000 ms/40 ms/90°, 64 × 64 acquisition matrix, 38 × 2.5 mm slices with a 0.5-mm gap) were acquired using a 1.5-T Siemens Avanto scanner (Erlangen, Germany) with a standard transmit/receive head coil and low specific absorption rate sequences ( ≤ 0.1 W/kg, head average) to reduce the risk of health hazards. One (for patients #2 and 4) or two (for patients #1, 3, 5, 6, 7, and 8) 10-min resting-state EPI time series (depending on patient comfort inside the scanner and time constraints) and T1-weighted structural scans were acquired.

### Intracranial EEG Pre-processing and IED Classification

icEEG recorded during fMRI was corrected offline for scanner-related artifact (37) and reviewed by expert users (UC and MC) to identify and classify all IED using BrainVision Analyzer2 (Brain Products GmbH, Germany) and compared with clinical long-term icEEG recording and reports.

**Figure 1:**
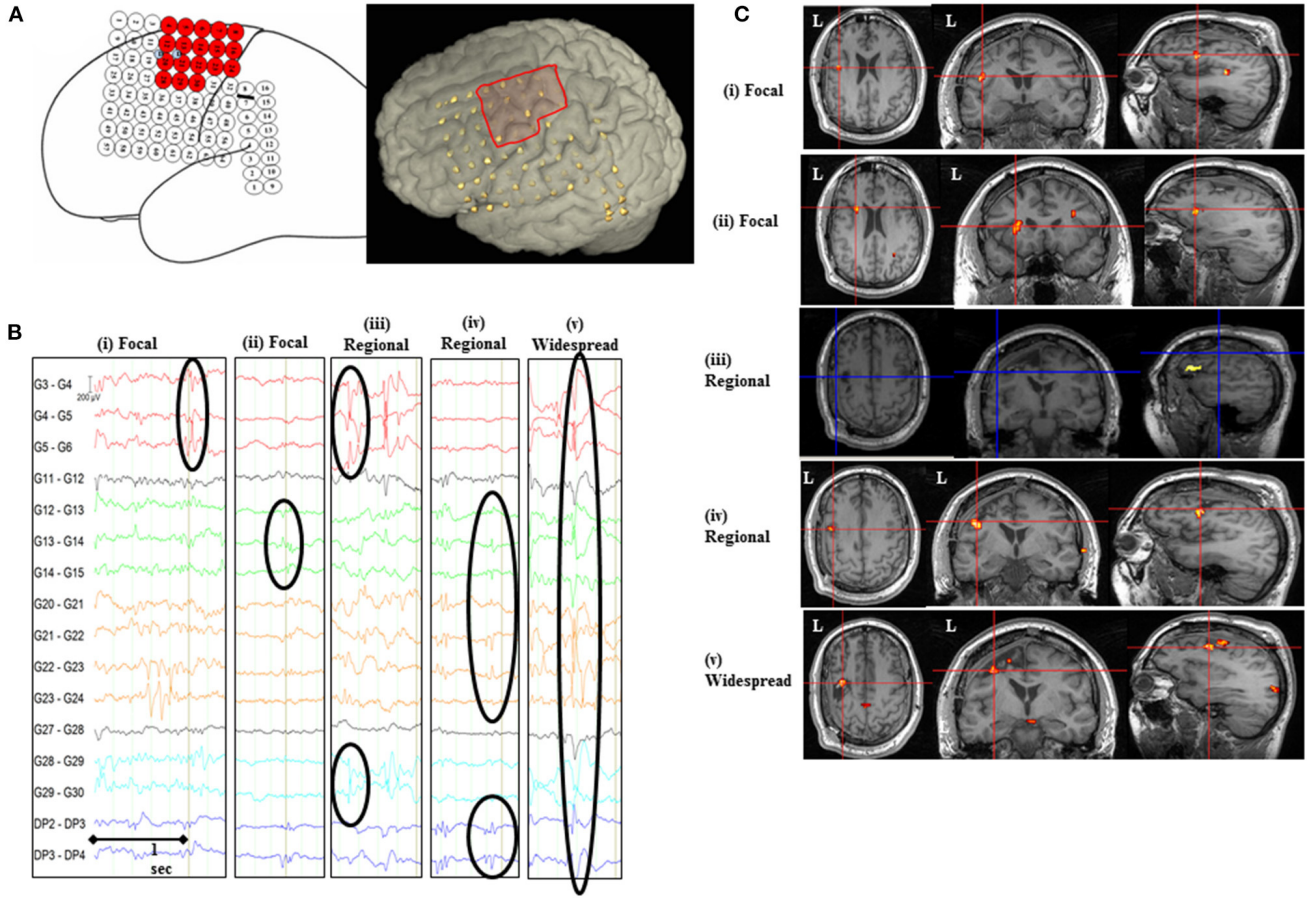
IED-related BOLD changes for Patient #2. **(A)** Implantation scheme of intracranial grid, strip, and depth electrodes and the invasively defined EZ is highlighted as red-colored electrodes on the sketched diagram and red square on the 3D rendered brain. **(B)** Representative sample of icEEG showing different IED classes based on their topographic distribution and localization included in the general linear model. **(C)** SPM[F] maps (*p* < 0.001) for different IED types overlaid on co-registered postsurgical T1-volume. (i) Focal IED [G4, 5 (*n* = 72); Concordant plus BOLD map]: changes in left inferior/middle frontal gyrus (GM cluster, within 2 cm of the surgical resection volume), left posterior temporal, and cuneus/precuneus. (ii) Focal IED [G12–15 (*n* = 29); BOLD map with Some concordance]: changes in left medial occipital (GM cluster), left inferior/middle frontal gyrus (within 2 cm of the surgical resection volume), left parieto-temporal, and cingulate gyrus. (iii) Regional IED [G4–6 + G12, 13 + G22–24 + G28–30 (*n* = 80); Discordant BOLD map]: changes in left inferior frontal gyrus (GM cluster 2.6 cm from the surgical resection volume), medial superior frontal gyrus, left temporo-occipital, and left middle frontal gyrus. (iv) Regional IED [G12–15 + G21–24 + DP2–4 (*n* = 350); Concordant plus BOLD map]: changes in left middle/inferior frontal gyrus (GM cluster, within 2 cm of the surgical resection volume), medial superior frontal gyrus, and supplementary motor area. (v) Widespread IED [G4–8 + G12–15 + G20–24 + G28–30 + DP2–4 (*n* = 244); Concordant plus BOLD map]: changes in left middle frontal gyrus (GM cluster, within 2 cm of the surgical resection volume), right middle temporal gyrus, posterior supplementary motor area, and cingulate/precuneus.

The identified IEDs were classified for the purpose of fMRI modeling according to the topographic distribution and localization (Table 2). For this topographic scheme, IED were classified according to the number of electrodes involved, their spatial location, field extent, and propagation (38) (Figure 1, Table 2) as either Focal: if they involved 2–4 contiguous electrode contacts and had similar field; Regional: if they involved 5–10 contiguous electrode contacts that may span up to two gyri; Widespread: if they involved more than 10 contiguous electrode contacts; or Non-contiguous: if they had a focal or regional field but also propagated to non-contiguous electrode contacts. The Regional, Widespread, and Non-contiguous classes taken together formed the non-focal IED class.

Furthermore, to assess our second hypothesis, we divided IEDs for their relationship with the surgical resection volume (i.e., confirmed EZ): IED classes overlapping the surgical resection volume were identified as IZ1 (primary irritative zone) and IED classes outside the surgical resection volume were identified as IZ2 (secondary irritative zone) (39) (see Table 2).

For patients #3 and #7 one of the two icEEG-fMRI sessions had to be excluded: for patient #3, icEEG had scanning-related artifacts and patient #7 had a subclinical seizure during one of the sessions (40).

### fMRI Processing and Modeling

The fMRI data were analyzed using Statistical Parametric Mapping 8 (www.fil.ion.ucl.ac.uk) after discarding the first two volumes to account for the T1-saturation effect. Functional imaging data were corrected for slice acquisition time, realigned to the mean, and spatially smoothed using an isotropic Gaussian kernel of 8-mm FWHM (41).

A general linear model (GLM) was built to map IED-related hemodynamic changes. For patients who underwent two EPI series, these were included in a single GLM as separate sessions. Each IED was represented either as a zero-duration stick function (individual IED) or blocks (runs of IED). Each IED class was modeled as a separate effect and the corresponding time series of stick functions or blocks convolved with the canonical hemodynamic response function and its temporal and dispersion derivatives. In line with previous analyses, 24 inter-scan realignment parameters [6 realignment parameters from image pre-processing and a Volterra-expansion of these (42)] were included in the GLM as confounds to account for motion-related effects similar to our previous work (40).

### Assessment of IED-Related BOLD Changes

For each IED class, the presence of significant BOLD clusters was assessed over whole brain using SPM[F]-maps at a statistical threshold of *p* < 0.001 (uncorrected for family-wise error) and a cluster threshold of five contiguous voxels, as in previous studies from our group and others (15, 22, 26, 32, 40, 43). The resulting SPMs were co-registered with pre- and post-surgical T1-weighted MRI scans using rigid-body registration in SPM. The localization of BOLD clusters, for each IED class, was visually assessed in relation to the surgical resection volume. Clusters of activity were also assessed in remote areas including cuneus, precuneus, cingulate gyrus, medial frontal lobe, and other brain areas such as basal ganglia, thalamus. The fitted BOLD time course for each cluster was plotted and classified as increases, decreases, or biphasic (consisting of both increases and decreases) according to the sign of the peak change relative to baseline.

The concordance of BOLD maps with surgical resection volume (i.e., confirmed EZ) was assessed for each IED class on icEEG using a concordance classification scheme in line with our previous work (15, 22, 26, 40, 43) as either:

- Entirely concordant: All BOLD clusters overlapping with/or located within 2 cm of the surgical resection volume in the same lobe.
- Concordant plus: The global statistical maximum BOLD cluster (GM cluster) overlapping with/or located within 2 cm of the surgical resection volume in the same lobe and other clusters were remote (i.e., >2 cm away in different lobe or opposite hemisphere) from the surgical resection volume.
- Some concordance: The GM cluster was remote from the surgical resection volume and at least one of the other clusters overlapped with or located within 2 cm of the surgical resection volume in the same lobe.
- Discordant: all clusters were remote, i.e., more than 2 cm from the surgical resection volume in the same lobe or were in a different lobe or opposite hemisphere from the surgical resection volume.

BOLD clusters confined to the ventricular system, vascular tree, edges, and base of brain and cerebellum were not considered further in this analysis (20, 43–46).

We performed chi-square tests (χ^2^) (SPSS Statistics) to assess the association between (1) topographic IED classes and level of concordance of BOLD maps/presence of BOLD clusters in remote brain areas/presence of balloon cells in patients with FCD; (2) IZ1/IZ2 IED classes and level of concordance of BOLD maps/presence of BOLD clusters in remote brain areas/presence of balloon cells in patients with FCD; (3) presence of balloon cells in FCD patients and level of concordance of BOLD maps.

## Results

The clinical details for eight patients fulfilling selection criteria are summarized in Table 1. There were six males; the median age at the time of icEEG-fMRI was 32 years and the median age at seizure onset was 7.5 years. Six patients had frontal lobe epilepsy, one had temporal lobe epilepsy, and one had parietal lobe epilepsy. The median follow-up time with ILAE class I/II postsurgical outcome was 6 years.

### Classification of IED

All patients had a mixture of different topographic IED classes (Table 2). The number of IED classes in any given patient ranged between 3 and 7 (median 4.5). There was a total of 38 IED classes across the group. Out of these 38 IED classes, 22 were Focal, and 16 were non-focal: Regional = 9, Widespread = 2, and Non-contiguous = 5 according to topographic classification scheme (see Table 2). In terms of irritative zones, 29/38 IEDs originated from IZ1 (Focal = 18, Regional = 8, Widespread = 2, and Non-contiguous = 1) and 9/38 from IZ2 (Focal = 4, Regional = 1, Widespread = 0, and Non-contiguous = 4).

### Distribution of IED-Related BOLD Changes for IED Classes

All IED classes were associated with significant BOLD clusters (Table 3) that were both co-located with recording electrodes but also in regions remote from them (Figure 1). BOLD clusters were seen within the surgical resection volume (Concordant) in 71% (27/38) of IED-related BOLD maps. The cluster of concordance corresponded to the statistical global maxima in 8 maps (Entirely Concordant = 1, Concordant plus = 7) and to the second or other significant cluster in 19 maps (Some concordance). At least two maps were concordant in every patient, with a mean of 70% of the maps per patient being concordant (range 50–100%).

**Table 3:** BOLD changes for individual IED class.

**Patient ID #**	**IED type**	**BOLD clusters (↑increase**, **↓decrease**, ****↑↓**biphasic)**									**Level of concordance**
		**Neocortex**								**Other Remote BOLD clusters**	
		**Right**				**Left**					
		**Frontal**	**Parietal**	**Temporal**	**Occipital**	**Frontal**	**Parietal**	**Temporal**	**Occipital**		
1	PSMA1–3	↓MFG	**↓Superior parietal**			↓IFG				↑**↓Medial SFG**	SC
	ASMA1–3	↓MFG, ↑**IFG**	↓Superior parietal			↓IFG	↓Superior parietal			↓SMA, Medial SFG, Precuneus/Cuneus. ↓Medial occipital	SC
	ASMA1–3 + PSMA1–3	↓SFG, MFG				↓**SFG**, MFG		↓ITG		↓Cuneus, Cingulate, Medial SFG/SMA	SC
	PC5, 6	↓MFG			↓Lateral superior					↓Medial SFG/SMA, Precuneus/Cuneus	SC
	PC5, 6 + AI5, 6	↓**SFG/MFG**,		↑Posterior temporal, MTG	↑Lateral superior	↓MFG		↑Posterior Temporal, MTG		↓Precuneus/Cuneus, Cingulate ↑SMA	C+
2	G4, 5					↓**IFG/MFG**		↑Posterior Temporal		↑Cuneus/Precuneus	C+
	G12–15					↓IFG/MFG	↓**Medial occipital**, Parieto-temporal			↓Cingulate, SMA	SC
	G4–6 + G12, 13 + G22–24 + G28–30					↓**IFG/**	↑MTG	↑Temporo-occipital		↑Medial SFG	D
	G12–15 + G21–24 + DP2–4					↓**MFG**/IFG				↑Medial SFG/SMA	C+
	G4–8 + G12–15 + G20–24 + G28–30 + DP2–4			↑MTG		↑**MFG**				↓SMA, Cingulate/Precuneus	C+
3	DA3, 4									↑**Thalamus**, ↓Cingulate	D
	G2 6, 14	↓OF	↓**Superior parietal**			↑MFG/IFG				↓Precuneus, Thalamus	SC
	DA3, 4 + G1 18, 27, 35, 43					↑**IFG**					EC
4	DA3–6	↓OF	↓Superior parietal	**↓ITG**, Temporo-occipital		↓IFG, OF			↓Medial occipital		SC
	DA4, 5 + GA51	↑IFG, SMC	**↓Superior parietal**	↑ITG, STG		↓OF/IFG			↑Medial occipital	↓Medial SFG	D
	DA2–6 + GA49–54	↑IFG	↓Inferior parietal	↑ITG		↓IFG				**↓Precuneus**	SC
5	FP2–4						***↑↓***Inferior Parietal			**↑Cingulate**	D
	FP2–4 + AM2–4	↑↓**FP**, SFG	↑↓Superior parietal								C+
	AM2–4 + FP1–4 PMFG3–6 + IFG9–11	↑**MFG**, OF								↑↓Precuneus, Medial SFG	SC
	AM1–4 + FP3–4 FP1–8 + AM1–14 + ASMA2–5+ PMFG3–10 + IFG5–10	↓MFG, OF				↑↓FP				↑↓**Medial SFG, Cuneus/**Precuneus	SC
	FP1–4 + AM1–6 FP1–8 + AM1–14 + FOF1–10 + ASMA2–7 + PMFG4–12 + IFG5–11		↓Temporo-parietal			↓FP, OF		↓ITG		↓**Cingulate**, Basal ganglia, Medial OF	SC
6	SF5–7									**↑Cingulate**	D
	GB4–6 + 14–16				↓Medial occipital		↓Superior parietal		↓Medial occipital	↓**Precuneus**, Medial SFG	D
	GC5–16					↑**SFG/MFG**				↓Cingulate, Medial SFG	C+
	SF5–7 + GB5–8 + GC5, 10, 11, 12, 15, 16					↑↓MFG				↑↓**Cingulate**, Thalamus	SC
7	LAH1, 2 + LPH1, 2 + LA3, 4	↑MFG	↑Superior parietal			↑IFG, MFG, OF	↑Superior parietal	↑**Posterior temporal**, STG		↑Precuneus, Cingulate, Medial SFG	SC
	LAH1–2		↑Superior parietal, posterior temporal				↑Superior parietal	↑**Posterior temporal**, STG		↑Precuneus, Cingulate, Medial SFG	SC
	LA3–4	↑SFG	↑Posterior temporal					↑Posterior temporal		↓**Precuneus**	D
	LPH 1–2		↑Superior parietal			**↑MFG**				↑**Cingulate**	D
	RA1, 2 + RH1, 2		↓**Superior parietal**, Temporo-parietal, temporo-occipital				↓Superior parietal			↓Precuneus, cingulate	D
	RA1, 2 + RAH1, 2 + LAH2, 3 + LPH2, 3	↑MFG	↑Superior parietal	Temporal pole, Medial temporal		↑MFG, OF	↑Superior parietal	↑**Posterior temporal**, STG, ITG		↑Precuneus, Cingulate, Medial SFG	SC
8	D1 + D2 + G31	↑MFG	**↑SMG**, ↑Superior parietal				↑Superior parietal	↑Insula		↑Thalamus	C+
	D1 3–4	↑MFG	↑SMG, ↑Deep parietal						↑Medial occipital	**↑Thalamus**	SC
	D2 5–6	↑IFG	↑SMG				**↑SMG**				SC
	G23	**↓IFG**									D
	G31	**↓Medial SFG**									D
	G36	↑MFG	↑SMG, ↑Superior parietal			↑MFG	↑SMG, ↑Superior parietal			↑**Medial SFG**, Thalamus	SC
	G38	↑MFG	↑SMG				**↑SMG**			↑Cingulate	SC

*R, right; L, left; NAL, not-applicable; SFG, superior frontal gyrus; MFG, middle frontal gyrus; IFG, inferior frontal gyrus; MTG, middle temporal gyrus; FP, frontopolar; SMG, supramarginal gyrus; ITG, inferior temporal gyrus; SMC, sensori-motor cortex; STG, superior temporal gyrus; SMA, supplementary motor area; OF, orbitofrontal; EC, entirely concordant; C+, concordant plus; SC, some concordance; D, discordant*.

*Clusters shown in bold are global statistical maximum clusters*.

All maps except one contained more than one BOLD cluster (see Table 3). Across the group, BOLD clusters were distributed in the ipsi/contralateral hemisphere remote cortical or other brain areas including precuneus, medial superior frontal gyrus, cingulate, basal ganglia, and thalamus in 32/38 (84%) of BOLD maps (see Table 3).

#### Relationship With IED Topographic Classification

BOLD maps for non-focal IEDs (Regional, Non-contiguous, and Widespread) were more commonly concordant with surgical resection volume (Entirely Concordant = 1, Concordant plus = 5, Some Concordance = 8; 14/16, 87.5%) than for Focal IEDs (Concordant plus = 2, Some Concordance = 11; 13/22, 59%), (χ^2^ = 7.08, *p* < 0.05). Presence of BOLD clusters in remote cortical and/or other brain areas, i.e., precuneus, medial frontal, cingulate, and thalamus, was more frequent for non-focal IED maps (15/16, 93%: Regional = 8, Non-contiguous = 5, and Widespread = 2) as compared to Focal IED maps (17/22, 77%) but the difference did not reach statistical significance.

#### Relationship With Irritative Zones

BOLD maps were concordant with the surgical resection volume for 20/29 (68%) IED classes from IZ1 (Entirely Concordant = 1; Concordant plus: 6; Some concordance: 13), and 7/9 (77%) IZ2 IED (Concordant plus: 1; Some concordance: 6). The map's level of concordance or presence of BOLD changes in remote cortical and/or other brain areas did not differ significantly between IZ1 and IZ2 IED classes.

#### Structural Abnormalities

Seven patients had structural abnormalities seen on MRI (Table 1). In six patients, these were in the *EZ* [focal cortical dysplasia (FCD) = 5, hippocampal sclerosis (HS) = 1], and one patient had an incidental finding of HS unrelated to the *EZ*.

In the subgroup of patients with FCD, four patients had FCD type IIB with balloon cells and one patient had FCD type IIA with no balloon cells (see Table 2). All patients with FCD showed at least one map with a BOLD cluster overlying the lesion. We did not find a statistically significant association between presence/absence of balloon cells and different IED classes and level of concordance of BOLD maps in this small subgroup of patients.

The patient with hippocampal sclerosis (#7) did not show any BOLD cluster directly overlying HS; however, BOLD clusters were seen within 2 cm of the structural abnormality.

## Discussion

Scalp EEG-fMRI studies have shown that IED-related BOLD changes in EZ can predict good postsurgical outcome (9, 13, 14, 22–25, 47–49). One of the limiting factors for these studies has been low sensitivity of scalp EEG to capture the whole spectrum of epileptiform activity that can be revealed by invasive recordings (50, 51). Therefore, what is considered the baseline (“non-epileptic” state) in scalp EEG-fMRI studies must in fact contain a significant amount of epileptic discharges. Simultaneous icEEG-fMRI allows us to overcome this problem by exploring whole-brain changes for epileptiform discharges recorded directly from the cortex using icEEG. This study revealed significant BOLD signal changes for a wide range of IEDs using simultaneous icEEG-fMRI. Furthermore, we found that:

- Significant BOLD clusters for IEDs on icEEG were localized both within the surgical resection volume and remote cortical and other brain areas;
- More than 70% of IED classes showed BOLD maps concordant with the surgical resection volume, where BOLD clusters were seen within the surgical resection volume;
- IED with wider topographic distribution and localization: non-focal IED classes on icEEG were associated with the presence of BOLD clusters within the surgical resection volume.

Previous studies using icEEG-fMRI have shown BOLD changes related to IEDs and seizures (32, 33, 40, 52, 53). Comparison of visual and automated IED classification on icEEG (53) presented a more objective interpretation of icEEG, but there was no statistically significant difference in concordance of the BOLD maps for two IED classification techniques. The relationship of BOLD clusters in surgical resection volume and in remote brain areas for different IED classes has not been explored in previous studies. For this study, we think that visual classification of IEDs based on their topographic distribution and localization and IZs, which reflects clinical insight of the expert user, facilitates clinical interpretation of resulting BOLD maps. We compared distribution of BOLD clusters in surgical resection volume (i.e., level of concordance) and remote brain areas for different IED classes using icEEG-fMRI in the largest group of patients to date who had undergone epilepsy surgery and had a good postsurgical outcome with a long follow-up time (median: 6 years). The surgical resected volume can be rendered confirmed EZ considering long postsurgical seizure freedom.

### Methodological Considerations

The feasibility and safety of simultaneous icEEG-fMRI has been established (27, 28, 30, 31). Signal degradation can be observed within up to 1 cm (often less at 1.5 T as in this study) of the electrode contacts and is orientation dependent (27), therefore limiting interpretation of the BOLD maps in the electrode contact's immediate vicinity. However, BOLD effects are generally more widespread (27, 54). BOLD maps revealed significant clusters for different IED classes on icEEG, which were concordant with the surgical resection volume (confirmed EZ) and other non-invasive and invasive investigations, and were also seen in distant areas known to be related to resting-state networks associated with interictal discharges (55, 56). Therefore, it is unlikely that these changes are false positive. Also, icEEG has high sensitivity to show IEDs from smaller generators as compared to scalp EEG (50, 51) and, thus, may be associated with relatively weaker BOLD changes from smaller brain regions; this is in line with previous icEEG-fMRI studies (29, 32, 33, 40).

IEDs were represented as single or series of events in separate regressors for each different class to evaluate specific BOLD pattern in a GLM framework (12, 22, 24, 32) using a standard hemodynamic response function and its derivatives as a hemodynamic kernel (41) to account for a degree of variability in hemodynamic peak delay and duration (12, 24).

In patients with a single seizure onset zone, there may be more than one IED class reflecting different topographic localization and distribution and IZs and not all of these require removal for good surgical outcome. Our interpretation of the IED classes took into account spatial localization, field distribution, propagation, and their relationship with the EZ (1, 38, 39).

In the concordance classification scheme, the first two levels of concordance—Entirely concordant and Concordant plus—are defined based on the location of GM cluster overlapping with or within 2 cm of the surgical resection volume ± presence of other BOLD clusters. For BOLD maps with Some concordance, a cluster other than GM cluster was overlapping with or within 2 cm of the surgical resection volume. In this retrospective study, confirmed EZ was known, and this other cluster in the surgical resection volume was identified. However, during prospective pre-surgical evaluation of patients with a presumed EZ, this cluster can be identified by a consensus agreement, for example, if it is concordant with the structural lesion such as FCD and/or other non-invasive/invasive localization techniques. Our choice of 2 cm as a distance threshold (within a single lobe) to ascertain concordant BOLD clusters reflects the uncertainties associated with implantation and co-registration-related brain shift and the anticipated spatial dislocation of two classes of signals due to neurovascular coupling (57, 58). We evaluated the level of concordance of IED-related BOLD maps irrespective of sign of BOLD change, as both BOLD increases and decreases can be found in the EZ (20, 22, 59, 60).

### Neurophysiological and Neurobiological Significance

BOLD clusters were seen in multiple areas for all IEDs on icEEG, and these areas included surgical resection volume (i.e., confirmed EZ) and adjacent/remote apparently healthy cortex. This suggests the possibility of common underlying brain areas or networks recruited as propagation nodes or even generators for different IED classes (50, 51, 61–63), or these widespread BOLD changes may be secondary to extensive underlying pathology (64). We suggest that BOLD changes in cortex and other brain areas remote from the surgical resection volume (i.e., confirmed EZ) may represent propagated epileptic activity in agreement with scalp EEG-fMRI (16, 22, 24, 65, 66) and electric source imaging studies (67). Also, this propagated epileptic activity in remote cortical or other brain areas such as precuneus, medial frontal, cuneus, and thalamus may represent an interaction with resting-state networks in line with previous scalp EEG-fMRI studies (55, 56), which can have implications on level of cognition and consciousness (55, 68) at some level and grants further research. We noted that changes in these areas that are part of default mode network were deactivations, but activations were also seen for some IEDs (see Table 3). Though a complete picture of underlying neuronal activity for IEDs may not be seen on icEEG (48) due to its limited spatial sampling, it is difficult to further elucidate whether these activations represent propagation of epileptic activity and deactivations represent involvement of default mode network. Future investigations correlating IEDs on icEEG with topographic maps of IEDs on scalp EEG and their associated BOLD changes will be required to understand the full pathologic nature of such networks.

The presence of BOLD clusters in surgical resection volume (confirmed EZ), as reflected by level of concordance of BOLD maps, was associated with topographic and field distribution of IED on icEEG. Non-focal IEDs on icEEG with wider topographic and field distribution (Regional, Widespread, and Non-contiguous) showed BOLD clusters in surgical resection volume more commonly, compared to focal IEDs on icEEG. This finding is similar to a recent scalp EEG-fMRI study (66) in which widespread epileptic discharges were more likely to show BOLD activation in seizure onset areas. The significance of this finding raises interesting questions about the BOLD effect, for example: is there a spatial scale of neural activity below which the strength of the BOLD change reflects only the local intensity of that activity, in contrast to its spatial extent? Hemodynamic changes may be limited to the activation of a minimum neuronal volume and its synchronization on EEG (69); this may explain the more common presence of BOLD changes in surgical resection volume and remote areas for IEDs with more widespread field extent. It is possible that signal dropout in the local vicinity of icEEG contact (28, 29) can limit to show BOLD change for IED with a very focal field extent. Future imaging sequence development with less signal dropout around implanted electrodes may be able to localize BOLD changes for very focal IEDs on icEEG. In addition, duration of underlying field potentials for epileptic discharges reflected by the sharp wave width can also affect amplitude of the BOLD signal (70), and event parameterization (amplitude, frequency content and duration) may be a useful way forward to further investigate BOLD changes for IED on icEEG.

### Clinical Significance

We found that icEEG-fMRI has greater sensitivity: all patients showed IED-related BOLD changes, whereas previously published scalp EEG-fMRI studies have shown IED-related BOLD changes in 30–78% of patients (12, 24, 26). We suggest that this partly reflects the high sensitivity, specificity, and spatial resolution of icEEG (when placed judiciously) compared to scalp EEG (1) and the possibility that this results in more accurate definition of the BOLD baseline. Also, there could possibly be selection bias; patients with a clear focus and IEDs on scalp EEG are more likely to proceed for invasive icEEG.

The strength of our data is that the surgical resection volume represents confirmed EZ as reflected by long seizure freedom after surgery (1). This level of confidence is lacking in previous studies. We found BOLD clusters located in the surgical resection volume in 70% of the maps for different IED classes on icEEG. As icEEG-fMRI can reveal BOLD network across the whole brain and does not suffer from limited spatial sampling of icEEG, it is possible that BOLD clusters remote from surgical resection volume may represent other generator or propagator areas of epileptic activity that are not covered by icEEG. Small sample size and heterogenous underlying pathology could be considered limitations of this study, restraining generalized application of these findings to all patients undergoing epilepsy surgery. It will be interesting to compare in the future, in larger sample size, if there is any difference of BOLD patterns for IEDs on icEEG between seizure-free patients and patients who did not achieve seizure freedom after epilepsy surgery, and if it can inform epilepsy surgery approach.

In conclusion, icEEG-fMRI studies constitute a significant step toward the better understanding of hemodynamic changes related to epileptic activity. It can provide localization of BOLD network at whole-brain level with high sensitivity for different classes of interictal discharges on icEEG originating from focal areas. In addition, BOLD clusters in surgical resection volume (confirmed EZ) were seen more commonly for non-focal epileptiform discharges on icEEG.

## Data Availability Statement

The datasets presented in this article are not readily available because subjects consent forms explicitly mention all information will be kept secure and strictly confidential, accessible only to those involved directly in this research. No information will be passed to any third parties or outside the EU for any reason without explicit consent of the subjects. Requests to access the datasets should be directed to Prof. Louis Lemieux, louis.lemieux@ucl.ac.uk.

## Ethics Statement

The studies involving human participants were reviewed and approved by Joint research ethics committee of the National Hospital for Neurology and Neurosurgery, Queen Square, London (UCLH NHS Foundation Trust) and UCL Institute of Neurology, Queen Square, London, UK. The patients/participants provided their written informed consent to participate in this study.

## Author Contributions

UC, MC, and DC made substantial contributions to the conception, design, data collection, data analysis, interpretation of results, and writing the article. BD made substantial contributions to the recruitment of patients, data interpretation, and revising the manuscript critically for important intellectual content. MW made substantial contributions to the data interpretation and revising the manuscript critically for important intellectual content. JD made substantial contributions to the recruitment of patients, interpretation of results, and revising the manuscript critically for important intellectual content. LL made substantial contributions to the conception, design, data analysis, and revising the manuscript critically for important intellectual content. All authors contributed to the article and approved the submitted version.

## Funding

This work was partly funded through grants and bursaries from the Medical Research Council (MRC Grant No. G0301067), Action Medical Research, Swiss National Science Foundation (SNF grant 320030-141165 and 33CM30-140332, SPUM Epilepsy), University of Modena, Reggio Emilia, and UCL Institute of Neurology. This work was undertaken at UCLH/UCL, which received a proportion of funding from the Department of Health's NIHR Biomedical Research Centers funding scheme.

## Conflict of Interest

The authors declare that the research was conducted in the absence of any commercial or financial relationships that could be construed as a potential conflict of interest.

## Publisher's Note

All claims expressed in this article are solely those of the authors and do not necessarily represent those of their affiliated organizations, or those of the publisher, the editors and the reviewers. Any product that may be evaluated in this article, or claim that may be made by its manufacturer, is not guaranteed or endorsed by the publisher.
