# Supplementary material for: Mapping Epileptic Networks Using Simultaneous Intracranial EEG-fMRI
Source: Front Neurol. 2021 Sep 21;12:693504. doi: 10.3389/fneur.2021.693504 (PMC8490636; doi:10.3389/fneur.2021.693504)
Supplement: Supplementary file 1 [file Table_1.doc]

| **Table S1: Electrode implantation schemes** | |
| --- | --- |
| **Patient ID** | **Implantation scheme**  Electrode type - Number and arrangement of electrode contacts: Anatomical coverage (label used for icEEG-fMRI)* |
| 1 | Depth - 1x6: R anterior SMA (ASMA)  Depth - 1x6: R posterior SMA (PSMA)  Depth - 1x8: R anterior Insula (AI)  Depth - 1x8: R posterior Insula (PI)  Depth - 1x8: R anterior Cingulum (AC)  Depth - 1x10: R middle Cingulum (MC)  Depth - 1x10: R posterior Cingulum (PC) |
| 2 | Grid – 8x8: L fronto-parietal (G)  Grid – 2x8: L parieto-temporal (GA)  Depth – 1x4: L anterior SFG (DA)  Depth – 1x4: L posterior SFG (DP) |
| 3 | Grid - 8x8: L fronto-parietal (G1)  Strip - 1x6: L sup. fronto-polar  Strip - 1x6: L inf. fronto-polar  Depth - 1x6: L IFG anterior (DA)  Depth - 1x6: L IFG posterior (DP)  Grid - 8x2: L fronto-polar (G2) |
| 4 | Grid - 8x6: L frontal (GA)  Strip - 2x8: L precentral gyrus (GB)  Strip - 2x8: L postcentral gyrus (GC)  Strip - 1x6: L anterior orbitofrontal  Strip - 1x6: L posterior orbitofrontal  Depth - 1x6: L IFG superior (DA)  Depth - 1x6: L IFG inferior  Grid - 8x4: L fronto-temporal |
| 5 | Depth - 1x16: R anterior medial orbito-frontal (AM)  Depth - 1x16: R posterior medial orbito-frontal (AM)  Depth - 1x12: R fronto-polar (FP)  Depth - 1x10: R lat. floor of orbito-frontal (FOF)  Depth - 1x12: R anterior SMA (ASMA)  Depth - 1x12: R posterior SMA (PSMA)  Depth - 1x16: R posterior middle frontal gyrus (PMFG)  Depth - 1x12: R IFG (IFG) |
| 6 | Grid - 8x8 L frontal/parietal (GA)  Grid - 2x8 L frontal pole (GB)  Strip - 1x8 L superior frontal gyrus (SF)  Strip - 1x6 L inferior frontal gyrus  High-density grid L mesial frontal (GC) |
| 7 | Depth - 1x6: L anterior hippocampus (LAH)  Depth - 1x6: L posterior hippocampus (LPH)  Depth - 1x6: L amygdala (LA)  Depth - 1x6: R anterior hippocampus (RAH)  Depth - 1x6: R amygdala (RA) |
| 8 | Depth – 1x4: R anterior Supramarginal gyrus (D1)  Depth – 1x6: R Posterior Inferior Supramarginal gyrus (D2)  Depth – 1x6: R Sensori-motor cortex  Grid - 8 x 8 R parietal and superior temporal (G) |

*: Only electrodes with labels in brackets (e.g. “(G)”) were recorded from during icEEG-fMRI.
